# Association between Poststroke Depression and Psychological Crisis: A Retrospective Cross-Sectional Study

**DOI:** 10.1155/2021/6698521

**Published:** 2021-01-26

**Authors:** Han-Chin Hsieh, Pei-Jin Yang, Yu-Chi Huang, Yan-Yuh Lee, Tsung-Hsun Yang, Szu-Ying Wu, Po-Cheng Chen

**Affiliations:** ^1^Department of Physical Medicine and Rehabilitation, Kaohsiung Chang Gung Memorial Hospital, Chang Gung University College of Medicine, Kaohsiung, Taiwan; ^2^Department of Chinese Medicine, Kaohsiung Chang Gung Memorial Hospital, Chang Gung University College of Medicine, Kaohsiung, Taiwan; ^3^Department of Nursing, Meiho University, Pingtung, Taiwan; ^4^Department of Sports Medicine, Kaohsiung Medical University, Kaohsiung, Taiwan; ^5^Department of Public Health, College of Medicine, National Cheng Kung University, Tainan, Taiwan

## Abstract

**Objective:**

To investigate the association between poststroke depression (PSD) and psychological crisis in patients who have experienced a stroke within 6 months.

**Methods:**

This was a retrospective cross-sectional study that enrolled patients within 6 months after stroke onset. The investigators reviewed medical charts to obtain patients' baseline characteristics, and a psychologist evaluated each patient for depression using the Taiwanese Depression Questionnaire (TDQ) and for psychological crisis using the Triage Assessment System (TAS). A generalized linear model (GLM) was used to analyze the association between the results of the TDQ and TAS.

**Results:**

Ninety-seven patients with stroke were included. Age (*p* = 0.003), time since onset of stroke (*p* = 0.041), diabetes mellitus (*p* = 0.004), hypertension (*p* = 0.016), heart disease (*p* = 0.005), and TDQ score were significantly different between the hemorrhagic stroke group and the ischemic stroke group. The TDQ score was significantly lower in the hemorrhagic stroke group (*p* = 0.012). The TDQ score was associated with the TAS total score and each domain score, and the presence of heart disease was associated with poorer TAS score in the behavioral domain (*p* = 0.016).

**Conclusion:**

PSD is likely an important component of psychological crisis in stroke patients. For clinicians, a comprehensive psychologic evaluation is necessary to optimize treatment.

## 1. Introduction

Stroke remains a leading cause of mortality and disability worldwide [1, 2], and patients may suffer from neurologic sequela such as hemiplegia or paresis, language deficit, and impairment of activities of daily living [3–7]. In addition to neurologic and functional deficits, patients also encounter significant challenges in terms of their support system, alteration of self-esteem, change to self-image, and altered living standards [3, 7]. Therefore, these patients are prone to development of depressive mood, anxiety, denial behavior, insomnia, or other psychological comorbidities [5, 6, 8, 9] and have a poststroke depression (PSD) prevalence of 31.1% and a poststroke anxiety prevalence of 20.4% [10, 11]. An increased prevalence of emotional disturbance may lead to psychological crisis [9, 12], which can be defined as when the summation of stressors faced by an individual exceeds their capacity to cope [13]. A crisis state is characterized by affective disequilibrium, cognitive imbalance, and behavioral instability [14]. There is often a negative impact on an individual's mental health, which restricts their ability to partake in purposeful life activities [15].

According to the DSM-V, a current diagnosis of PSD was defined as mood disorders due to stroke with depressive features, a major depressive-like episode, or mixed-mood features. Patients have a depressed mood or loss of interest or pleasure along with at least two, but fewer than five, symptoms of major depression, lasting for 2 weeks or longer [16]. However, several factors hinder the diagnosis of depressive disorders in stroke patients, such as a lack of accurate screening tools, limited communication capability and emotional disturbance caused by lesion location in the brain [16, 17]. This may lead to undiagnosed and overlooked PSD [17]. On the other hand, psychologic crisis and the associated emotional disturbance can be assessed using the Triage Assessment System (TAS). The TAS describes an individual's reaction during a crisis in three domains, affective, behavioral, and cognitive [14, 18, 19]. Affective reactions include anger/hostility, anxiety/fear and sadness/melancholy, and range from a stable mood with normal variation of affect appropriate to daily functioning, to frequent negative mood, disturbance of concentration, or even decompensation or depersonalization [14].

Previous studies have demonstrated that depression has negative impacts on functional outcomes and rehabilitation progress in patients with stroke [20, 21]. However, the pathophysiology of PSD is poorly understood [21] and few studies have examined whether PSD is related to psychological crisis. It is not yet known whether psychological crisis could perturb a patient's coping strategy, inhibiting their ability to perform activities of daily living and their progression in further rehabilitative programs. To facilitate optimal functional recovery, timely detection of mood disorder enabling early psychological intervention is important. In this study, we aimed to investigate the association between PSD and psychological crisis in patients who have experienced a stroke within 6 months.

## 2. Methods

### 2.1. Study Design

This study was a retrospective cross-sectional survey.

### 2.2. Participants

Patients with less than 6 months of poststroke who were at least 20 years of age were enrolled from one rehabilitation ward of a medical center from August 2018 through January 2020. Rehabilitation physicians consulted psychiatrists to evaluate whether it was appropriate for a patient to be referred to a psychologist once the patient had sufficient communication skills to be able to answer the psychological questionnaires. Patients with an unknown stroke type classification were excluded. This study was approved by the Institutional Review Board of Chang Gung Memorial Hospital (IRB No. 202001548B0).

### 2.3. Data Source and Measurement

The investigators reviewed medical charts and extracted patient demographic characteristics, including age, sex, body mass index (BMI), stroke type, stroke lesion side, stroke lesion location, premorbid functional status, and comorbidities. Marital status, history of alcohol or drug abuse, and history of previous suicide attempt(s) were ascertained by the psychologist prior to psychological evaluation.

### 2.4. Psychological Evaluation

After a basic assessment and evaluation by a psychiatrist, enrolled patients diagnosed with depressive symptoms were referred to a psychologist for evaluation of the degree of symptoms and their reaction to crisis. The Taiwanese Depression Questionnaire (TDQ) [22] is a culture-sensitive depression screening tool that has been reported to be of good validity and internal consistency, and is reliable for use in a community sample. The questionnaire consists of 18 items, examining disturbances in mood, issues with sleeping, appetite and energy, interest in normal activities, crying, and feelings about the future. Patients recorded their responses on a 4-point Likert scale (ranging from 0 to 3) to indicate whether each item had been experienced after the onset of stroke; 0, 1, 2, and 3 points indicated that the symptom occurred for less than 1 day, 1-2 days, 3-4 days, and 5-7 days in a week, respectively.

The TAS for psychological crisis intervention [18] is used to understand patients' reactions to crisis events. This framework presumes that reactions to crisis are seen in three domains: affective, behavioral, and cognitive. A psychologist evaluated the patients' reactions along all three domains, and identification of the complex interactions among the three domains assists in eliminating protracted concerns regarding mental health. Reactions are rated on a scale of 1 to 10, with 10 indicating the most severe reaction. The intensity and direction of the crisis intervention would depend on the degree of severity on the TAS.

### 2.5. Statistical Analyses

The categorical variables in the ischemic and hemorrhagic stroke type groups were compared using the chi-square test or Fisher's exact test, while the continuous variables were compared using the independent *t*-test. After checking the normality of the TDQ and TAS using the Shapiro-Wilk test, Pearson's correlation analysis was employed to investigate the relationship between the outcomes of the TDQ and TAS (including total score and each domain score). A correlation coefficient of 0.10~0.30 was taken to represent a small association, 0.30~0.50 was considered a moderate correlation, and 0.50 or larger was taken to represent a large correlation. A generalized linear model (GLM) was used to analyze the associations between the outcomes of the TDQ and TAS (including total score and each domain score) when the demographic variables were controlled. In addition to stroke type, the demographic variables that were statistically significantly different between the stroke type groups were entered into the regression model. The assumptions of the GLM, such as multicollinearity, normality, linearity, homoscedasticity, and independence of the residuals, were checked before the regression model was conducted. A *p* value less than 0.05 was defined as indicating statistical significance. Statistical analyses were performed using SAS software, Version 9.4 of the SAS System for Windows (SAS Institute Inc., SAS Campus Drive, Cary, North Carolina 27513, USA).

## 3. Results

**Figure 1:**
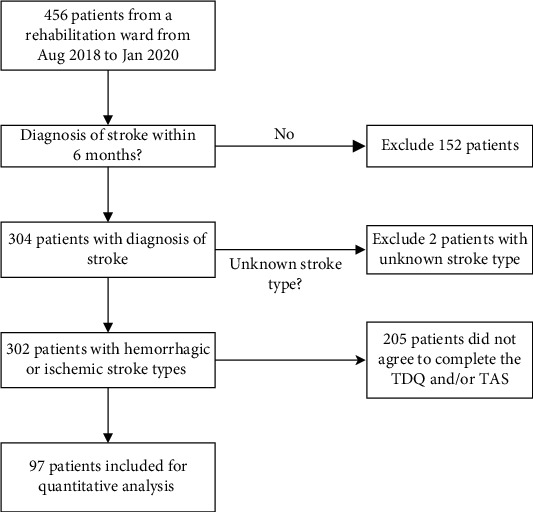
Flow diagram of patient selection.

In total, 456 patients were referred for psychological evaluation in one rehabilitation ward of a medical center from August 2018 through January 2020; 304 of whom had been diagnosed with stroke within the previous 6 months. Two patients with unknown stroke types were excluded from further analysis. As we aimed to investigate the relationship between depression and psychological crisis among stroke patients, only 97 patients with records of both TDQ and TAS implementations were included for quantitative analysis. A flow diagram of patient selection is shown in Figure 1.

**Figure 2:**
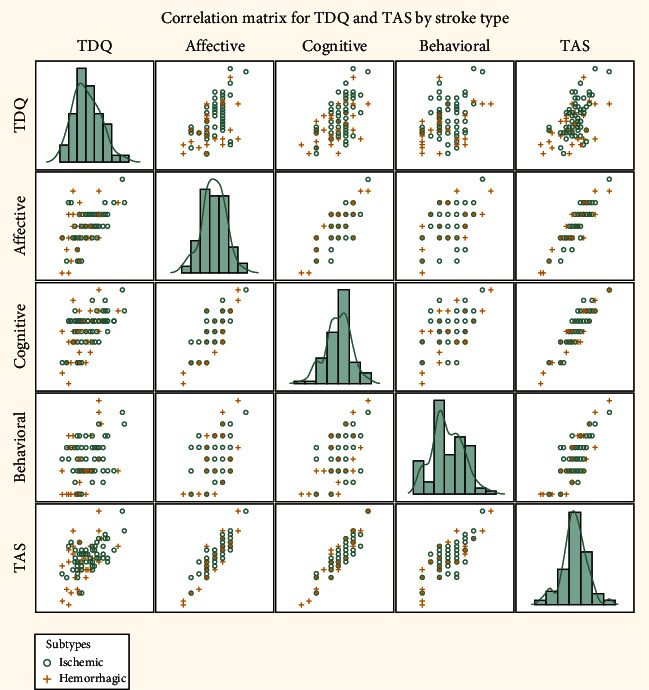
Correlation matrix for TDQ and TAS by stroke type.

The demographic characteristics of the patients are presented in Table 1. Statistically significant differences were noted in age (55.63 ± 11.22 versus 63.49 ± 11.65 years, *p* = 0.003), time since onset of stroke (83.10 ± 61.11 versus 58.09 ± 51.98 days, *p* = 0.041), diabetes mellitus (*p* = 0.004), hypertension (*p* = 0.016), and heart disease (*p* = 0.005) between the hemorrhagic stroke group and the ischemic stroke group. The scores on the TDQ and each domain of the TAS are presented in Table 2, and the TDQ score was statistically significantly lower in the hemorrhagic stroke group (12.17 ± 6.05 versus 15.57 ± 6.06, *p* = 0.012). The relationship between the scores of the TDQ and TAS is depicted in Figure 2 and Supplementary Table 1, and the effect size of the Pearson's correlation coefficient was large (*r* = 0.55 to 0.57) for the TDQ and TAS scores (with the exception of the behavioral domain, *r* = 0.35).

Stroke type, time since onset of stroke, sex, age, comorbidities (including diabetes mellitus, hypertension, and heart disease), and TDQ score were entered into the GLM in order to identify associations between the TAS score (including each domain score) and TDQ score. GLM analysis explained 38% of the variance of the TAS total score (*R*^2^ = 0.38). The TDQ score had a significant effect on the TAS total score after the demographic variables were controlled (*ϐ* = 0.48, SE = 0.08, *p* < 0.001) (Table 3). The TDQ score remained significantly associated with each domain of the TAS, and heart disease was significantly associated with a higher (worse) score in the TAS (*ϐ* = 1.04, SE = 0.42, *p* = 0.016) (Supplementary Table 2). The adjusted mean of the behavioral domain score among the patients with heart disease was significantly higher (worse) than in those without heart disease (5.12 versus 4.08, *p* = 0.016) (Table 4).

## 4. Discussion

The results of our study indicated a correlation between the Taiwanese Depression Questionnaire (TDQ) score and all domain scores and the total score of the Triage Assessment System (TAS) in patients with stroke. After adjusting all covariates between the two stroke groups, hemorrhage and ischemic, the TDQ score remained associated with the TAS score. In addition, the presence of heart disease was associated with a poorer TAS score.

Depression is believed to have a negative impact on stroke recovery [20, 21], quality of life, and functional independence [23–25]. Skidmore et al. [26] demonstrated that depressive symptoms and executive functions were significantly correlated with participation in rehabilitation in patients with stroke, and many studies have focused on a potential association between poststroke depression (PSD) and brain network dysfunction [3, 27–32]. Current mainstream treatment for PSD includes antidepressant medication, psychotherapy, and psychosocial support [16, 33–35]. However, aside from depression, patients with stroke may also encounter shock, confusion, denial, fear, and anxiety [9], which may not be adequately addressed by the aforementioned treatments. From a psychologic crisis point of view, the affective response represents one of the reactions when facing a crisis event. The TAS evaluates a patient's reaction to crisis in the affective, behavioral, and cognitive domains, which may further help to provide effective treatment in this population as these three domains are intricately linked [18]. Emotions influence the way people react to challenges and give rise to a coordinated set of behavioral, experiential, and physiological responses [36]. Affective and cognitive processes together shape behavior and decision-making [37, 38]. Recent imaging study indicated that stroke may lead to dysfunction in executive function areas of the brain, which induces behavioral and affective changes [39]. In this study, we identified that depression in stroke survivors is associated with a higher TAS score, and therefore may need to be considered in order to optimize treatment strategies, which currently might fall short of addressing psychological aspects after stroke [9, 13, 35, 40, 41].

In this study, we also found that the presence of heart disease was associated with a poorer behavioral domain TAS score in patients with stroke, and the adjusted mean of the behavioral domain score among those with heart disease was significantly higher than in those without. This result implied that patients with stroke and heart disease tended to have more immobility and exhibit avoidance behavior, which might essentially be related to impaired mobility and motivation. Previous studies also revealed poor energy and mobility limitations in patients with stroke and heart disease [42, 43]. Nevertheless, study of possible demographic factors and psychological crisis in patients with stroke is scarce, and larger-scale evaluations may be required to provide further clarity.

This was the first study to identify a relationship between PSD and psychological crisis in patients within 6 months of stroke onset. Though rarely discussed before, the role of psychological crisis during rehabilitation of stroke patients cannot be overlooked.

## 5. Limitations

There were some limitations of our study. First, the sample size was small, and all the participants were recruited from the same medical center. Selection bias may exist due to limited communication capabilities; in that, only those capable of expressing their emotions were able to be assessed. Second, more specific classification of the associated underlying disease could be performed with an adequate sample size. The potential existence of a multicollinear relationship between the emotional domain of the TAS and the TDQ score should also be considered. Finally, this was a cross-sectional study, and further larger-scale and long-term follow-up studies should be conducted to observe the temporal changes in psychological status in patients with stroke.

## 6. Conclusion

Depressive symptoms appear to be an important component of psychological crisis, as patient's behavioral, affective, and cognitive reactions are intricately connected in the face of a psychologic crisis. Our findings reflect the necessity for comprehensive psychologic evaluation in order to develop an optimized rehabilitation plan for patients who have experienced a stroke within 6 months.

## Figures and Tables

**Table 1:** Demographic characteristics of patients with hemorrhagic or ischemic stroke.

	Stroke type		*p* value
	Hemorrhagic	Ischemic	
	Mean ± SD (range)	Mean ± SD (range)	
Age (years)	55.63 ± 11.22 (36~82)	63.49 ± 11.65 (21~89)	0.003^∗∗^
BMI	25.28 ± 6.04 (17.78~44.98)	25.22 ± 3.47 (20.00~34.67)	0.960
Time since onset of stroke (days)	83.10 ± 61.11 (11~180)	58.09 ± 51.98 (9~180)	0.041^∗^
	*N*	*N*	*p* value
Sex			0.904
Female	15	31	
Male	15	36	
Marital status			0.165
Married	19	53	
Separated, divorced, or widowed	11	14	
Alcohol or drug abuse			0.663
No	29	62	
Yes	1	5	
Previous suicide attempt(s)			0.309
No	29	67	
Yes	1	0	0.904
Stroke lesion side			0.071
Both	0	8	
Left	15	22	
Right	15	37	
Stroke lesion location			0.237
Both	1	10	
Cortex	7	12	
Subcortex	22	45	
Premorbid functional status			0.173
Partially dependent	0	6	
Totally independent	30	61	
Diabetes mellitus			0.004^∗∗^
No	24	32	
Yes	6	35	
Hypertension			0.016^∗^
No	0	11	
Yes	30	56	
Kidney disease			0.597
No	25	52	
Yes	5	15	
Lung disease			0.096
No	30	60	
Yes	0	7	
Heart disease			0.005^∗∗^
No	28	44	
Yes	2	23	
Autoimmune disease			0.225
No	28	66	
Yes	2	1	
History of depression or psychiatric disorders			0.093
No	28	67	
Yes	2	0	
History of cognitive impairment			NA
No	30	67	
Yes	0	0	

SD: standard deviation; BMI: body mass index; NA: not assessed. ^∗^*p* value < 0.05, ^∗∗^*p* value < 0.01. The independent *t*-test was used for age and BMI. Fisher's exact test or the chi-square test was used for age and sex, marital status, alcohol or drug abuse, previous suicide attempt(s), diabetes mellitus, hypertension, kidney disease, lung disease, heart disease, autoimmune disease, history of depression or psychiatric disorder, history of cognitive impairment, stroke lesion side, and stroke lesion location.

**Table 2:** Psychological questionnaire results of patients with hemorrhagic or ischemic stroke.

	Stroke subtype		*p* value
	Hemorrhagic	Ischemic	
	(*N* = 30)	(*N* = 67)	
	Mean ± SD	Mean ± SD	
TDQ score	12.17 ± 6.05	15.57 ± 6.06	0.012^∗^
Total TAS score	14.63 ± 5.62	16.34 ± 3.52	0.133
Affective domain	5.60 ± 1.79	6.03 ± 1.35	0.195
Cognitive domain	5.47 ± 2.16	6.18 ± 1.47	0.108
Behavioral domain	3.57 ± 2.25	4.13 ± 1.52	0.216

SD: standard deviation; TDQ: Taiwanese Depression Questionnaire; TAS: Triage Assessment System. ^∗^*p* value < 0.05.

**Table 3:** Generalized linear model for analysis of TDQ and TAS scores.

Parameter	Estimate	SE	*t*	*p* value
Intercept	15.07	3.07	4.90	<0.001^∗∗∗^
Sex
Female	-0.10	0.94	-0.11	0.915
Male	Reference	—	—	—
Stroke type
Hemorrhagic	0.77	1.29	0.60	0.553
Ischemic	Reference	—	—	—
Time since onset of stroke (days)	-0.01	0.01	-1.10	0.274
Diabetes mellitus
Yes	-0.59	1.04	-0.57	0.571
No	Reference	—	—	—
Hypertension
Yes	-2.39	1.59	-1.50	0.137
No	Reference	—	—	—
Heart disease
Yes	2.57	1.14	2.25	0.027^∗^
No	Reference	—	—	—
Age	0.04	0.04	0.89	0.377
TDQ	0.48	0.08	6.20	<0.001^∗∗∗^

*R* ^2^ = 0.38, VAR_coefficient_ = 20.34, Mean_total TAS score_ = 22.21. TDQ: Taiwanese Depression Questionnaire; TAS: Triage Assessment System; VAR: variance. ^∗^*p* value < 0.05, ^∗∗^*p* value < 0.01, ^∗∗∗^*p* value < 0.001.

**(a):** 

Heart disease	Adjusted mean of affective domain	*p* value^a^
Yes	6.18	0.242
No	5.79	

**(b):** 

Heart disease	Adjusted mean of cognitive domain	*p* value^a^
Yes	6.49	0.190
No	6.02	

**(c):** 

Heart disease	Adjusted mean of behavioral domain	*p* value^a^
Yes	5.12	0.016^∗^
No	4.08	

^a^Bonferroni adjustment for multiple comparisons. ^∗^*p* value < 0.05.

## Data Availability

The data is not available at the current stage because we are still conducting a secondary analysis.

## Supplementary Materials

Supplementary Materials Supplementary Table 1: correlation matrix of each correlation coefficient (*r* value) for TDQ and TAS outcomes by stroke type. Supplementary Table 2: generalized linear model for analysis of TDQ score and scores on each domain of the TAS.
