# Supplementary material for: Association between Poststroke Depression and Psychological Crisis: A Retrospective Cross-Sectional Study
Source: Biomed Res Int. 2021 Jan 26;2021:6698521. doi: 10.1155/2021/6698521 (PMC7857865; doi:10.1155/2021/6698521)
Supplement: Supplementary Materials — Supplementary Table 1: correlation matrix of each correlation coefficient (r value) for TDQ and TAS outcomes by stroke type. Supplementary Table 2: generalized linear model for analysis of TDQ score and scores on each domain of the TAS. [file 6698521.f1.docx]

Supplementary Table 1. Correlation matrix of each correlation coefficient (r-value) for TDQ and TAS outcomes by stroke type.

P-values for all coefficients < 0.01.

|  | **TDQ** | **Affective** | **Cognitive** | **Behavioral** | **TAS** |
| --- | --- | --- | --- | --- | --- |
| **TDQ** | 1.00 | 0.55 | 0.57 | 0.35 | 0.56 |
| **Affective** | 0.55 | 1.00 | 0.81 | 0.52 | 0.89 |
| **Cognitive** | 0.57 | 0.81 | 1.00 | 0.52 | 0.90 |
| **Behavioral** | 0.35 | 0.52 | 0.52 | 1.00 | 0.80 |
| **TAS** | 0.56 | 0.89 | 0.90 | 0.80 | 1.00 |

P-values for all coefficients < 0.01.

Abbreviations: TDQ, Taiwan Depression Questionnaire; TAS, Triage Assessment System.

Supplementary Table 2. Generalized linear model for analysis of TDQ score and scores on each domain of the TAS.

| **Affective domain** | | | | |
| --- | --- | --- | --- | --- |
| **Parameter** | **Estimate** | **SE** | **t** | **p-value** |
| **Intercept** | 3.55 | 0.88 | 4.02 | <0.001*** |
| **Sex** |  |  |  |  |
| Female | 0.05 | 0.27 | 0.17 | 0.866 |
| Male | reference | - | - | - |
| **Stroke type** |  |  |  |  |
| Hemorrhagic | 0.10 | 0.37 | 0.27 | 0.789 |
| Ischemic | reference | - | - | - |
| **Time since onset of stroke (days)** | <0.01 | <0.01 | -0.23 | 0.819 |
| **Diabetes mellitus** |  |  |  |  |
| Yes | -0.27 | 0.30 | -0.90 | 0.370 |
| No | reference | - | - | - |
| **Hypertension** |  |  |  |  |
| Yes | 0.01 | 0.46 | 0.02 | 0.985 |
| No | reference | - | - | - |
| **Heart disease** |  |  |  |  |
| Yes | 0.39 | 0.33 | 1.18 | 0.242 |
| No | reference | - | - | - |
| **Age** | 0.01 | 0.01 | 0.64 | 0.525 |
| **TDQ** | 0.13 | 0.02 | 5.75 | <0.001*** |
| R^2^=0.37, VAR_coefficient_=24.01, Mean_Emotion_=5.96 | | | | |
| **Cognitive domain** | | | | |
| **Parameter** | **Estimate** | **SE** | **t** | **p-value** |
| **Intercept** | 2.67 | 0.97 | 2.74 | 0.007** |
| **Sex** |  |  |  |  |
| Female | 0.12 | 0.30 | 0.40 | 0.688 |
| Male | reference | - | - | - |
| **Stroke type** |  |  |  |  |
| Hemorrhagic | 0.25 | 0.41 | 0.62 | 0.534 |
| Ischemic | reference | - | - | - |
| **Time since onset of stroke (days)** | <0.01 | <0.01 | -1.44 | 0.155 |
| **Diabetes mellitus** |  |  |  |  |
| Yes | -0.07 | 0.33 | -0.21 | 0.831 |
| No | reference | - | - | - |
| **Hypertension** |  |  |  |  |
| Yes | -0.35 | 0.50 | -0.69 | 0.491 |
| No | reference | - | - | - |
| **Heart disease** |  |  |  |  |
| Yes | 0.48 | 0.36 | 1.32 | 0.190 |
| No | reference | - | - | - |
| **Age** | 0.02 | 0.01 | 1.68 | 0.098 |
| **TDQ** | 0.16 | 0.02 | 6.31 | <0.001*** |
| R^2^=0.37, VAR_coefficient_=24.01, Mean_Cognition_=5.96 | | | | |
| **Behavioral domain** | | | | |
| **Parameter** | **Estimate** | **SE** | **t** | **p-value** |
| **Intercept** | 3.52 | 1.13 | 3.11 | 0.003** |
| **Sex** |  |  |  |  |
| Female | -0.11 | 0.35 | -0.31 | 0.759 |
| Male | reference | - | - | - |
| **Stroke type** |  |  |  |  |
| Hemorrhagic | 0.19 | 0.48 | 0.40 | 0.689 |
| Ischemic | reference | - | - | - |
| **Time since onset of stroke (days)** | <0.01 | <0.01 | -0.74 | 0.459 |
| **Diabetes mellitus** |  |  |  |  |
| Yes | -0.14 | 0.38 | -0.36 | 0.720 |
| No | reference | - | - | - |
| **Hypertension** |  |  |  |  |
| Yes | -0.95 | 0.59 | -1.62 | 0.110 |
| No | reference | - | - | - |
| **Heart disease** |  |  |  |  |
| Yes | 1.04 | 0.42 | 2.47 | 0.016* |
| No | reference | - | - | - |
| **Age** | <0.01 | 0.02 | -0.10 | 0.922 |
| **TDQ** | 0.09 | 0.03 | 3.16 | 0.002** |
| R^2^=0.20, VAR_coefficient_=42.05, Mean_Behavior_=3.96 | | | | |

*p-value < 0.05, **p-value < 0.01, ***p-value < 0.001

Abbreviations: TDQ, Taiwan Depression Questionnaire; TAS, Triage Assessment System.
